# Supplementary material for: A single-cell transcriptomic atlas characterizes the silk-producing organ in the silkworm
Source: Nat Commun. 2022 Jun 9;13:3316. doi: 10.1038/s41467-022-31003-1 (PMC9184679; doi:10.1038/s41467-022-31003-1)
Supplement: Supplementary file 1 — Supplementary Information [file 41467_2022_31003_MOESM1_ESM.pdf]

Supplementary Information

A single-cell transcriptomic atlas characterizes the silk-producing organ in the silkworm

Yan Ma[1], Wenhui Zeng[1], Yongbing Ba[2], Qin Luo[1], Yao Ou[1], Rongpeng Liu[1], Jingwen Ma[1], Yiyun Tang[1], Jie Hu[1], Haomiao Wang[1], Xuan Tang[2], Yuanyuan Mu[1], Qingjun Li[1], Yuqin Chen[1], Yiting Ran[1], Zhonghuai Xiang[1], Hanfu Xu[1]*

Affiliations:

[1] State Key Laboratory of Silkworm Genome Biology, College of Sericulture, Textile and Biomass Sciences, Southwest University, Chongqing, 400715, China.

[2] Shanghai OE Biotech. Co., Ltd., Shanghai, 201212, China

These authors contributed equally: Yan Ma, Wenhui Zeng, Yongbing Ba

Correspondence to: xuhf@swu.edu.cn (H.F.X.)

This PDF file includes:

Supplementary Figs. 1 to 7

Supplementary Tables 1 to 2

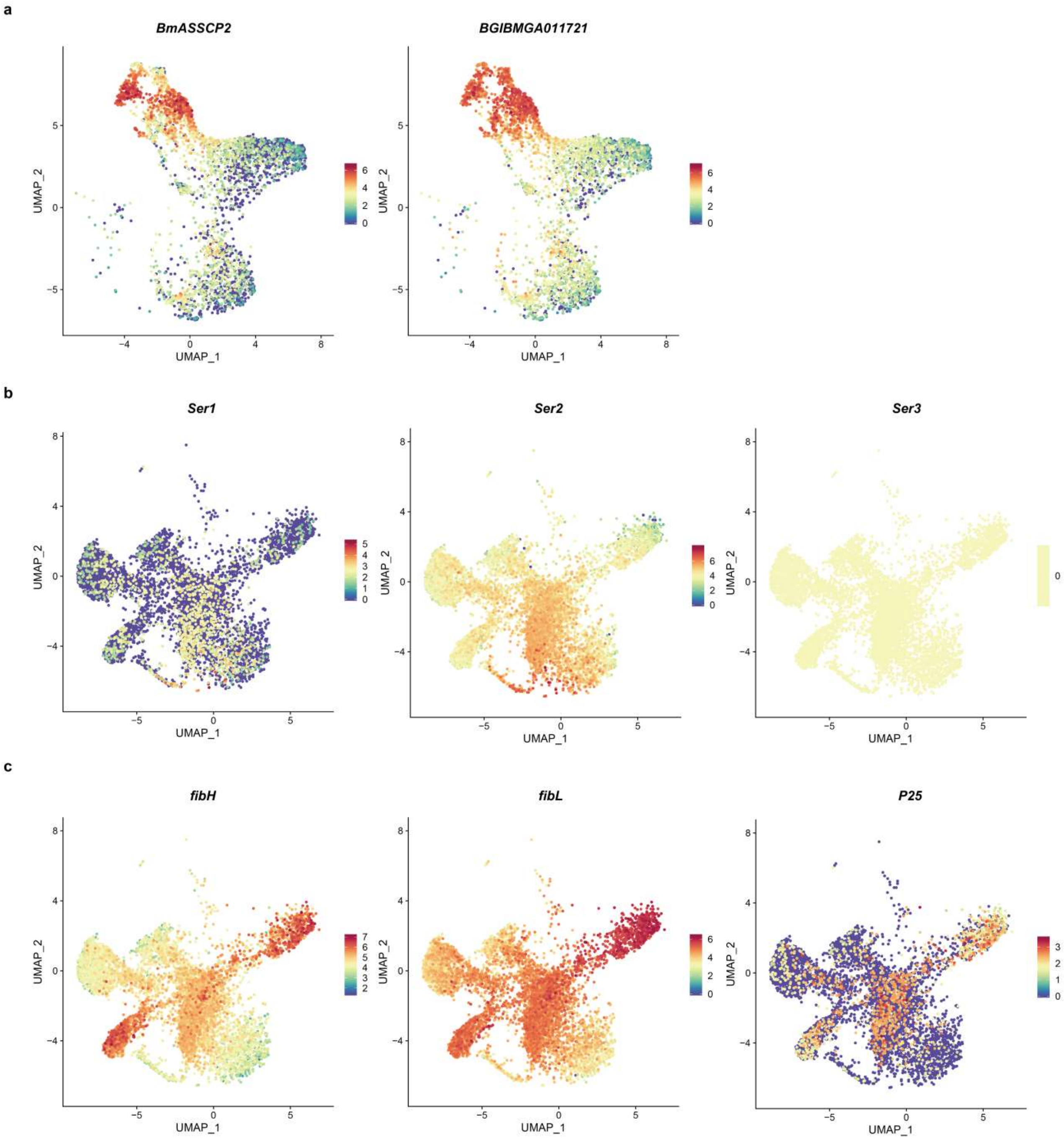

Supplementary Fig 1. Featureplots of the expression patterns of known classic marker genes. (a) Featureplots of the ASG marker genes *BmASSCP2* and *BGIBMGA011721*. (b) Featureplots of the MSG marker genes *Ser1*, *Ser2*, and *Ser3*. (c) Featureplots of the PSG marker genes *fibH*, *fibL*, and *P25*.

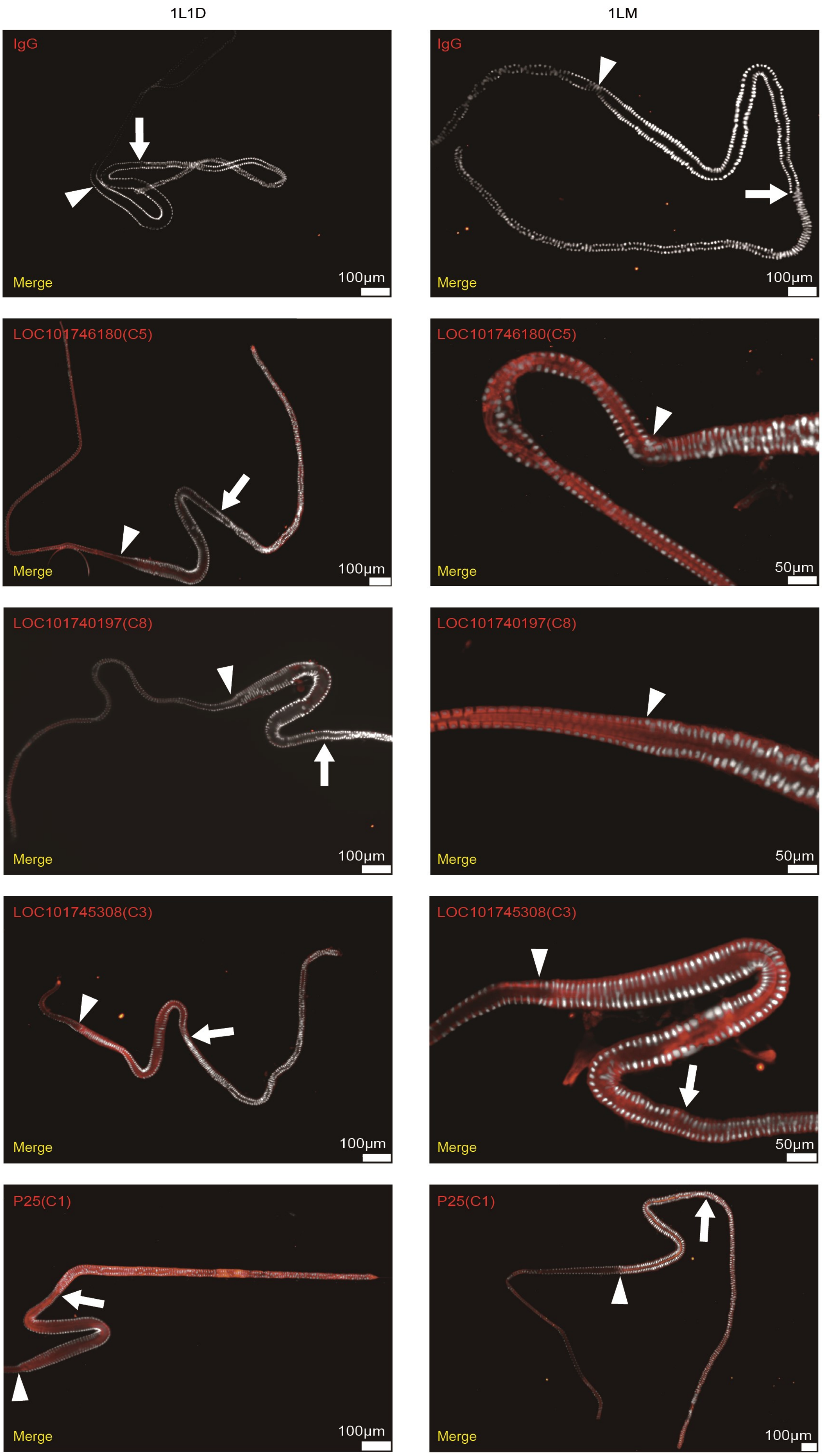
1L1D
1LM
IgG
Merge
100μm
IgG
Merge
100μm
LOC101746180(C5)
Merge
100μm
LOC101746180(C5)
Merge
50μm
LOC101740197(C8)
Merge
100μm
LOC101740197(C8)
Merge
50μm
LOC101745308(C3)
Merge
100μm
LOC101745308(C3)
Merge
50μm
P25(C1)
Merge
100μm
P25(C1)
Merge
100μm

Supplementary Fig 2. Immunofluorescence staining for selected representative marker genes in 1L1D and 1LM. The spatial expression pattern of these specific marker genes is shown in red, nuclei were stained with DAPI (white), the arrowhead (white) indicates the boundary between the ASG and MSG, and the arrow (white) indicates the boundary between the MSG and PSG. IgG was used as a negative control.

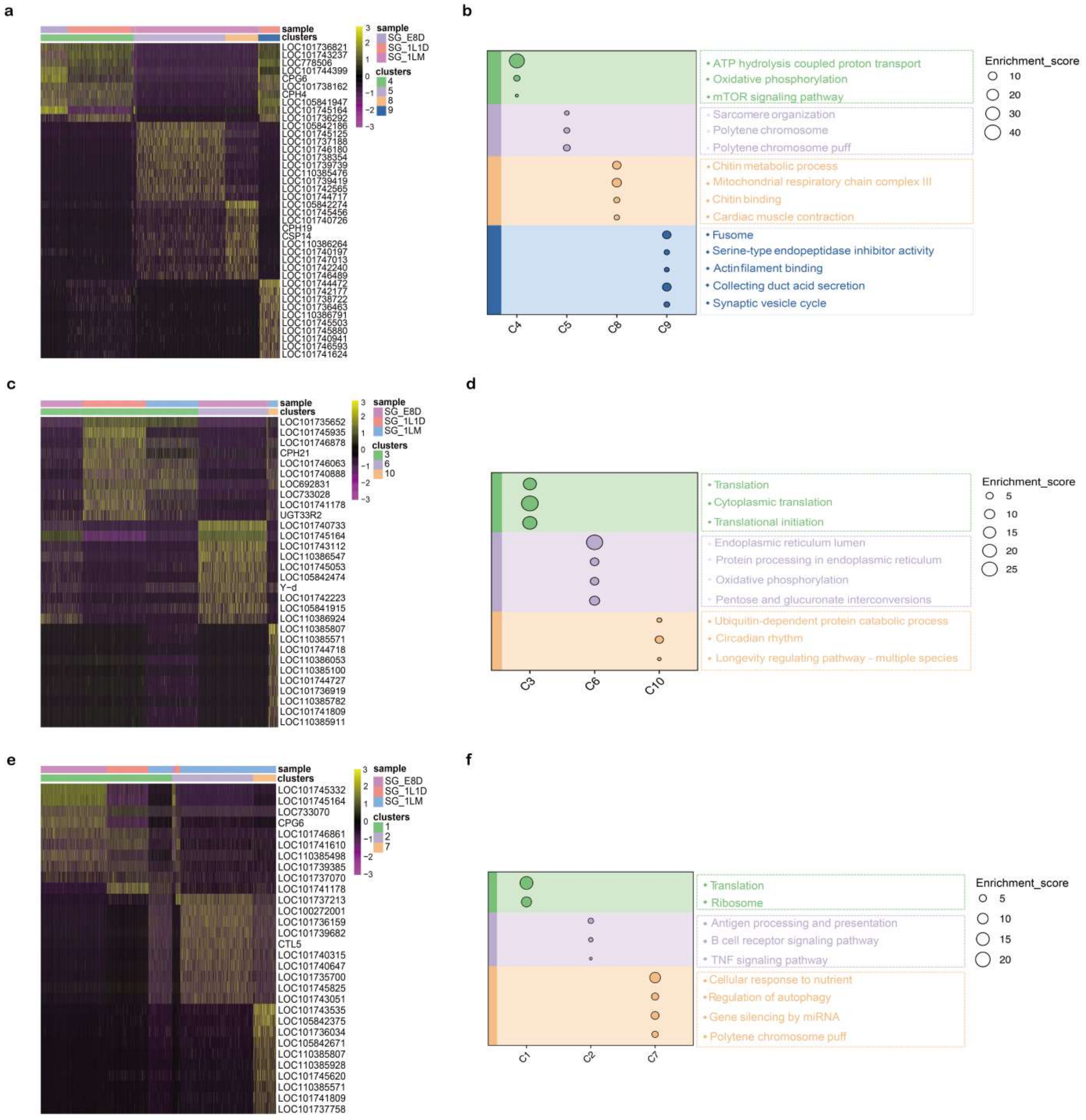

Supplementary Fig 3. Spatiotemporal distribution of the top 10 marker genes of each cell type in the ASG/MSG/PSG. Heatmap showing the distribution of the highly expressed genes in each cell type in E8D (a), 1L1D (c), and 1LM (e). Representative terms identified by performing an enrichment analysis of each cell type in the ASG (b), MSG (d) and PSG (f).

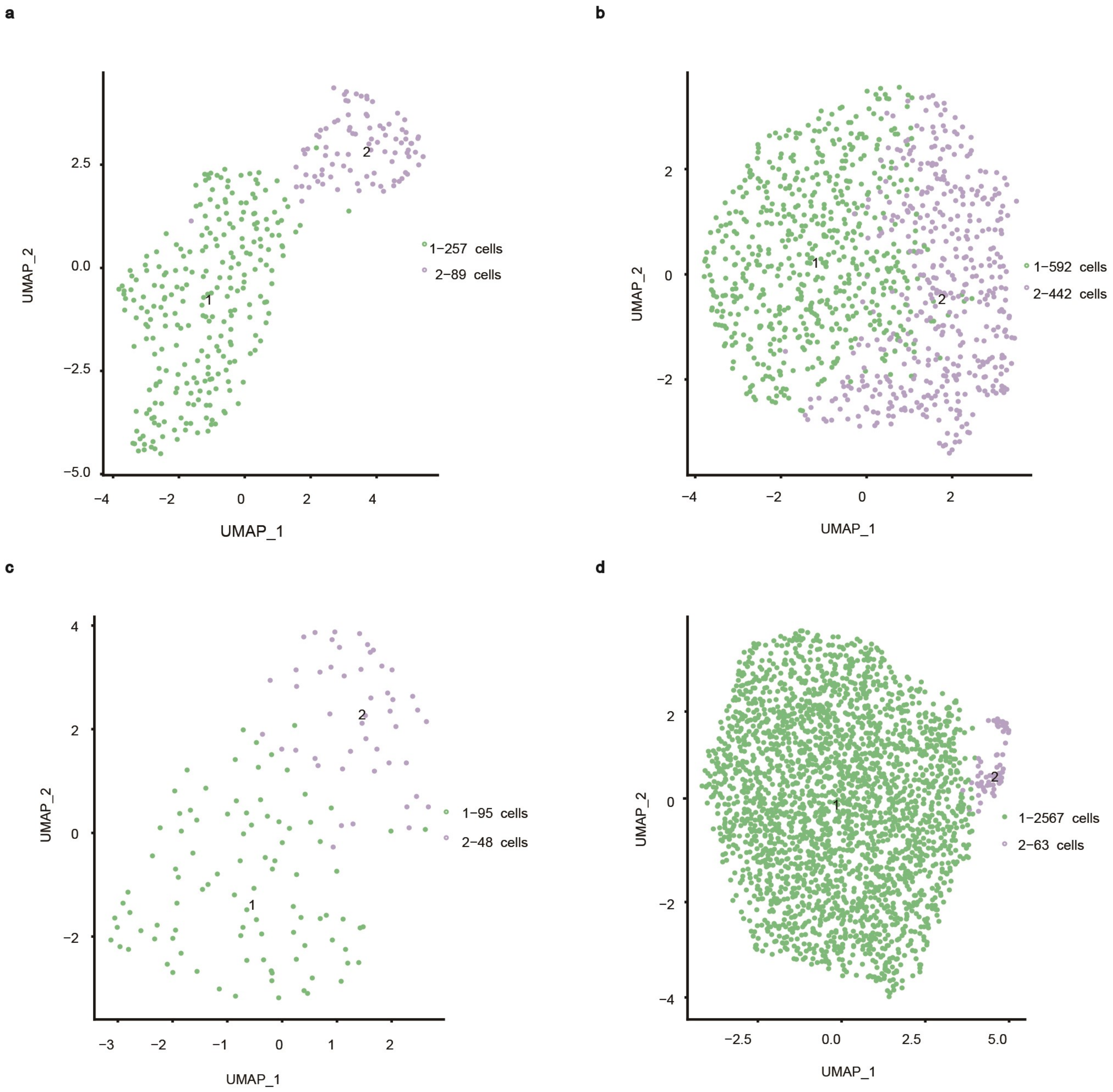

Supplementary Fig 4. Subcluster analysis of C9 (a), C6 (b), C10 (c), and C2 (d).

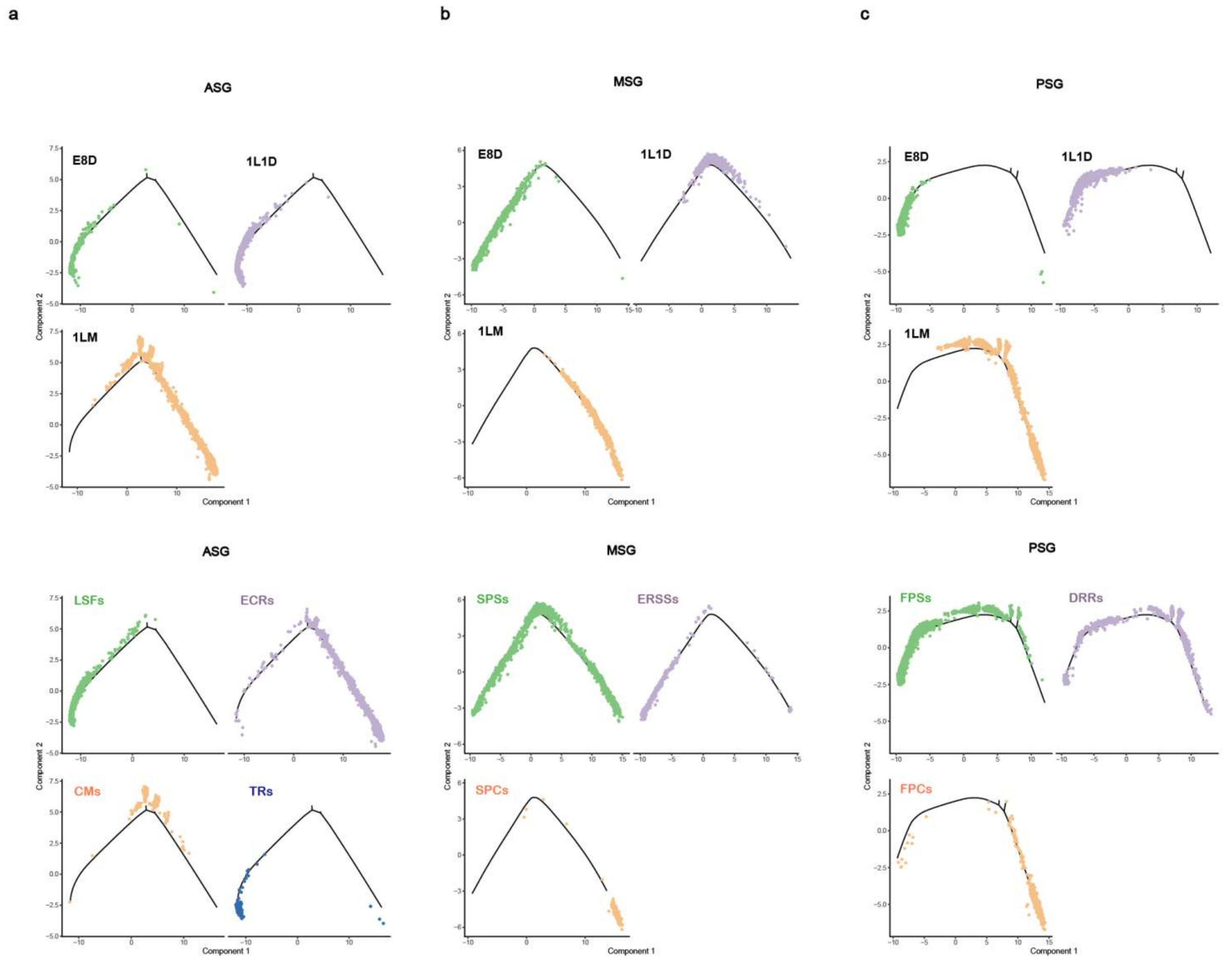

Supplementary Fig 5. Distribution of cells in the ASG (a), MSG (b), and PSG (c) along the pseudotime trajectory.

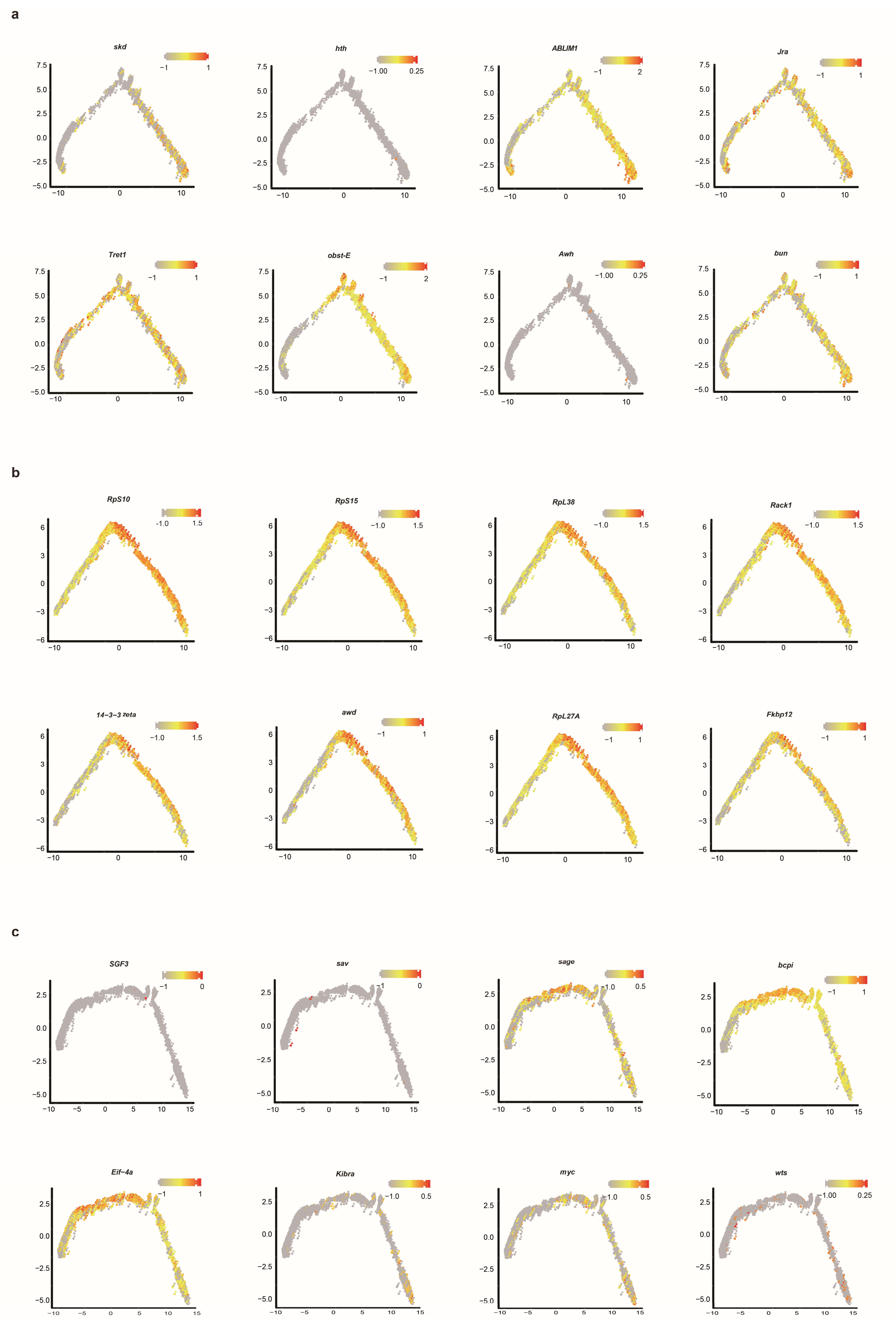
a
skd
hth
ABLIM1
Jra
Tret1
obst-E
Awh
bun
b
RpS10
RpS15
RpL38
Rack1
14−3−3 zeta
awd
RpL27A
Fkbp12
c
SGF3
sav
sage
bcpi
Eif−4a
Kibra
myc
wts

Supplementary Fig 6. Trajectory along the pseudotime progression of representative genes that activated and inactivated genes in ASG (a), MSG (b), and PSG (c) cells. The X-axis represents Component 1, and the Y-axis represents Component 2. The formula of this trajectory plot is log10 (value + 0.1).

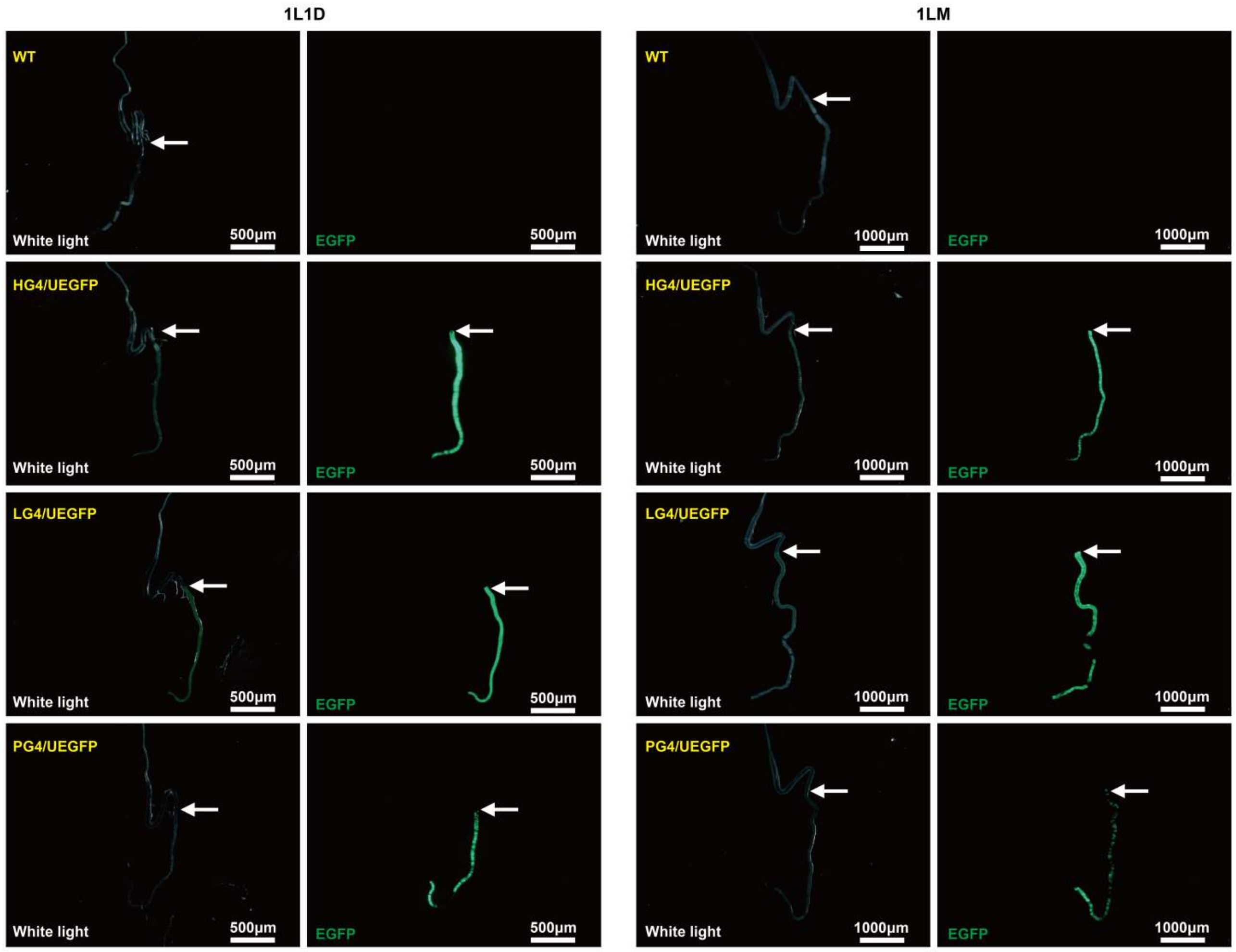

Supplementary Fig 7. Spatiotemporal expression of silk protein genes in the SG of GAL4/UAS transgenic silkworms at 1L1D and 1LM. Green fluorescence shows the distribution of EGFP expression driven by HG4, LG4, and PG4. The white arrow shows the boundary between the MSG and PSG.

Supplementary Table 1. Primer sequences used for qRT–PCR analysis

Name	Forward primer sequence (5' - 3')	Reverse primer sequence (5' - 3')
BmASSCP2	GCTTATGGGAGCGTTGTTC	TCCATTGCGTTCTTCTTGTTGA
BGIBMGA011721	GGTCGTCACTGCCACAAGC	CGTAGCGAGCGTATGTAGGAA
LOC101746861	GGCAAAAAGGTGGAATGGA	AAATCGCAAACAAAAGGAAGAATG
LOC101745308	TGGAGGTTCCAATTCGAGC	ACGCAGATAAGATACGGAGTTTC
Ser1	TCCACAACCGATAAGACGAGC	TTTCCTTGACCAGGCTTGTGT
Ser2	CCAACGATAATGATAAGCAATAC	TCCCCATCAGTGCTCTTCTCTAC
Ser3	TACAGGTATGGCTGCGGA	TCATCGGAGTCCTCGTCAT
Ser4	ACATTCGCATTCTTCCCACA	GCTGAAGCGAATAAACCGTA
LOC101743237	TCAGCACTAGCAACACCCAAA	ACCACACACCCCAATAGAAGG
LOC105841947	ATTGGAGGGTTTCAAGAGCAG	GTAATCGTTTATTGCCTCTCGG
LOC101746180	ATTGACCCGAGTAGACCGC	TTCTCCCCTAAAACCGCAA
fibH	CAGGGGATACGGACAAGGT	TTCACACAAGGCAGTGCTCT
fibL	GGAGGTGGAAGAATCTATGAC	TGTAGGCAGCGATGTTGT
P25	GGGTCTGCCCATCTTCCAC	CTCGCCAGCCAGTTCCTCT
LOC101740733	ATTCTTTTTGTCGCTTTGGC	CTTCATCGTCATCTTCTCCTTTT
LOC101743535	CGTAAACTCAGAGCGGCGG	AGACACCCAAACGATTCCATTC
LOC101740197	GGAACCTCTATCAACACCCG	CGGGGTTACAGAAGGGTTG
Btl	GTTGCTATGGGAGATAATGACGC	TGGTTGGCTGTGACCGTAAG
LOC101744718	TGGGATTTTATGGGGAACG	AACAACAGAGGGCAGCGAG
BmeIF4A	TTCGTACTGGCTCTTCTCGT	CAAAGTTGATAGCAATTCCCT

Supplementary Table 2. Antibodies used for immunofluorescence staining

Name of antibody	Form and species of antibody	Dilution
fibH	Polyclonal antibody, rabbit source	1:150
fibL	Polyclonal antibody, rabbit source	1:150
P25	Polyclonal antibody, rabbit source	1:150
Ser1	Polyclonal antibody, rabbit source	1:150
Ser2	Polyclonal antibody, rabbit source	1:150
Ser3	Polyclonal antibody, rabbit source	1:150
LOC101746180 (C5)	Polyclonal antibody, rabbit source	1:150
LOC101740197 (C8)	Polyclonal antibody, rabbit source	1:150
LOC101745308 (C3)	Polyclonal antibody, rabbit source	1:150
Btl (C9)	Polyclonal antibody, rabbit source	1:150
Anti-rat IgG (H+L), (Alexa Fluor® 555 Conjugate)	CST, #4417	1:150
Normal Rabbit IgG	CST, #2729	1:150

The polyclonal antibodies used in this manuscript are commercial antibodies synthesized by Zoonbio Biotechnology Co., Ltd., which is located in Nanjing, Jiangsu Province, China (homepage: http://zoonbio.bioon.com.cn/).
