# Supplementary material for: A single-cell transcriptomic atlas characterizes the silk-producing organ in the silkworm
Source: Nat Commun. 2022 Jun 9;13:3316. doi: 10.1038/s41467-022-31003-1 (PMC9184679; doi:10.1038/s41467-022-31003-1)
Supplement: Supplementary file 3 — Description for Additional Supplementary Files [file 41467_2022_31003_MOESM3_ESM.pdf]

Description of Additional Supplementary Files

File Name: Supplementary Data 1

Description: All marker genes for each cluster.

File Name: Supplementary Data 2

Description: Top 5 marker genes for each cluster.

File Name: Supplementary Data 3

Description: Specific marker genes for each cluster.

File Name: Supplementary Data 4

Description: Representative terms enriched in each cluster and subcluster analysis of Clusters 2, 6, 9 and 10.

File Name: Supplementary Data 5

Description: The pseudotime heatmap gene module of the top 10 genes in each cluster in the SG.

File Name: Supplementary Data 6

Description: All switching genes detected in ASG cells.

File Name: Supplementary Data 7

Description: All switching genes detected in MSG cells.

File Name: Supplementary Data 8

Description: All switching genes detected in PSG cells.

File Name: Supplementary Data 9

Description: Representative switching genes detected in the SG.

File Name: Supplementary Data 10

Description: Trajectory along the pseudotime progression of representative genes in SG cells.

File Name: Supplementary Data 11

Description: Representative marker genes regulating the development of SGs.

File Name: Supplementary Data 12

Description: Enrichment analysis of representative marker genes.

File Name: Supplementary Data 13

Description: Normalized data grouped by cluster.

File Name: Supplementary Data 14

Description: Representative GO terms for SPSs, ERSSs, and SPCs.

File Name: Supplementary Data 15

Description: Top 20 KEGG pathways for FPSs, DRRs, and FPCs.

File Name: Supplementary Data 16

Description: Detailed information of gene annotations.

File Name: Supplementary Software 1

Description: Single-cell RNA-seq analysis of SG_code

File Name: Source Data

Description: Source data of Fig. 3a and Fig. 6b
